# Copy number variations and constitutional chromothripsis (Review)

**DOI:** 10.3892/br.2020.1318

**Published:** 2020-06-26

**Authors:** Aldina Brás, António Sebastião Rodrigues, José Rueff

**Affiliations:** Centre for Toxicogenomics and Human Health (ToxOmics), Genetics, Oncology and Human Toxicology, NOVA Medical School, Faculty of Medical Sciences, NOVA University of Lisbon, Lisbon 1169-056, Portugal

**Keywords:** copy number variations, constitutional chromothripsis, congenital disease

## Abstract

Both copy number variations (CNVs) and chromothripsis are phenomena that involve complex genomic rearrangements. Chromothripsis results in CNVs and other structural changes. CNVs are frequently observed in the human genome. Studies on CNVs have been increasing exponentially; the Database of Genomic Variants shows an increase in the number of data published on structural variations added to the database in the last 15 years. CNVs may be a result of replicative and non-replicative mechanisms, and are hypothesized to serve important roles in human health and disease. Chromothripsis is a phenomena of chromosomal rearrangement following chromosomal breaks at multiple locations and involves impaired DNA repair. In 2011, Stephens *et al* coined the term chromothripsis for this type of fragmenting event. Several proposed mechanisms have been suggested to underlie chromothripsis, such as p53 inactivation, micronuclei formation, abortive apoptosis and telomere fusions in telomere crisis. Chromothripsis gives rise to normal or abnormal phenotypes. In this review, constitutional chromothripsis, which may coexist with multiple *de novo* CNVs are described and discussed. This reviews aims to summarize recent advances in our understanding of CNVs and chromothripsis, and describe the effects of these phenomena on human health and birth defects.

## 1. Introduction

Following the complete sequencing and analysis of the human genome, Snijders *et al* (1) assembled microarrays for genome-wide measurement of DNA copy numbers and CNVs of the normal human genome have been increasingly described (2). Genomic architecture has an important role in CNVs (3). Pericentromeric and sub-telomeric regions are rich in highly homologous duplicated segments of DNA >1 Kb in length and with >90% sequence similarity known as segmental duplications (SDs) (4). Non-allelic homologous recombination (NAHR) may serve a more prominent role in larger CNVs and SDs than smaller CNVs (5). SDs are also referred to as low-copy number repeats (LCRs) (4). Harel and Lupski (6) defined LCRs as clusters of paralogous sequences organized in hierarchical groups of direct and inversely orientated sequences. LCRs vary in copy number and also mediate CNV formation. Furthermore, LCR/SD pairs can contribute to initiate NAHR and result in the formation of CNVs. NAHR events between different chromatids give rise to duplications or deletions, whereas NAHR events on the same chromatid give rise to deletions (3).

Chromothripsis derived from chromosome (*chromo*) shattering (*thripsis*) is a phenomenon that involves complex chromosomal rearrangements (7). Marcozzi *et al* (7) reviewed a model of chromothripsis formation in which chromosomes are initially broken into small chromosomal fragments, and subsequently, the chromosomal fragments are reassembled into a new chromosome. However, the order and orientation of the fragments are altered compared with the structure of the original chromosome, and some of the chromosomal fragments may be lost as they are not incorporated during the reassembly process (7). Thus, chromothripsis is characterized by extensive genomic rearrangements and an oscillating pattern of DNA copy number levels of one or a few chromosomes (8). Chromothriptic breakpoints often occur in the vicinity of clusters of point mutations, termed kataegis (from the Greek for ‘thunderstorm’) (9), thus leading to the hypothesis that kataegic regions of hypermutations may indicate, or even lead to structural rearrangements (10).

## 2. CNVs

CNVs consist of duplications, deletions and insertions of DNA sequences into an individual's genome that range in size from 50 base pairs to millions of bases (11). They are structural variants that may involve complex genomic rearrangements and seem to possess additional mutations around their breakpoints (12). CNVs are seemingly frequent in the human genome. Zarrei *et al* (4) constructed a CNV map of the human genome and identified 11,742 CNV regions in the stringent map of healthy individuals. Aberrant pairing between mismatched copies of the segmental duplication and unequal crossing over in meiosis may be involved in deletions. Additionally, interstitial duplications may be due to inter- or intrachromosomal recombination between copies of the segmental duplications (13). Large LCRs are predisposed to DNA rearrangements, namely deletions, duplications and inversions, via NAHR. NAHRs give rise to recurrent structural variants, which share the same genomic content and size in unrelated individuals (14). Nonrecurrent structural variants which possess unique genomic content and size at a given locus in unrelated individuals are formed by other molecular mechanisms that include replicative and non-replicative mechanisms, reviewed in (15). LCRs may possess a dual role in structural variation: Mediating recurrent structural variants as substrates for NAHR and then stimulating non-recurrent variants via replication-based mechanisms (14). Gu *et al* (16) suggested that the high concentration of Alu elements in a specific region served as a suitable substrate for formation of CNVs and Alu-Alu-mediated mechanisms contribute considerably to the formation of complex CNVs.

The formation of structural variants may be associated with the genomic architecture. For example, NAHR- and nonhomologous-mediated CNVs are associated with different timings of DNA replication: Hotspots of NAHR-mediated events were enriched in early-replicating regions, whereas nonhomologous hotspots were enriched in late-replicating regions (17).

Recently, Hattori *et al* (18) reported a case with several features of the multiple *de novo* CNVs (mdnCNVs), including multiple rearrangements in perizygotic cells, non-recurrent rearrangements, and rearrangements that were present in one chromosomal arm but were not present at the inter-chromosomal translocation (18). According to Liu *et al* (19), the timeframe of the mdnCNV phenomenon predicts that the dnCNVs which occur prior to the embryo reaching the 2-cell stage are constitutional by default.

CNVs remain a major challenge with regard to clinical interpretation. The American College of Medical Genetics established standards and guidelines for interpretation and reporting of postnatal constitutional CNVs (20). There are three main categories of significance: Pathogenic, benign and uncertain clinical significance. The last category is subdivided into; likely pathogenic, likely benign and uncertain clinical significance (no sub-classification). The interpretation of the clinical relevance of CNV is complex but necessary to the practice of medicine.

## 3. Chromothripsis

Chromothripsis, chromoanasynthesis and chromoplexy are collectively termed chromoanagenesis (from the Greek *chromo* for chromosome and *anagenesis* for rebirth). For a recent review see Zepeda-Mendoza and Morton, 2019(21). In the present review, only chromothripsis will be discussed.

**Figure 1:**
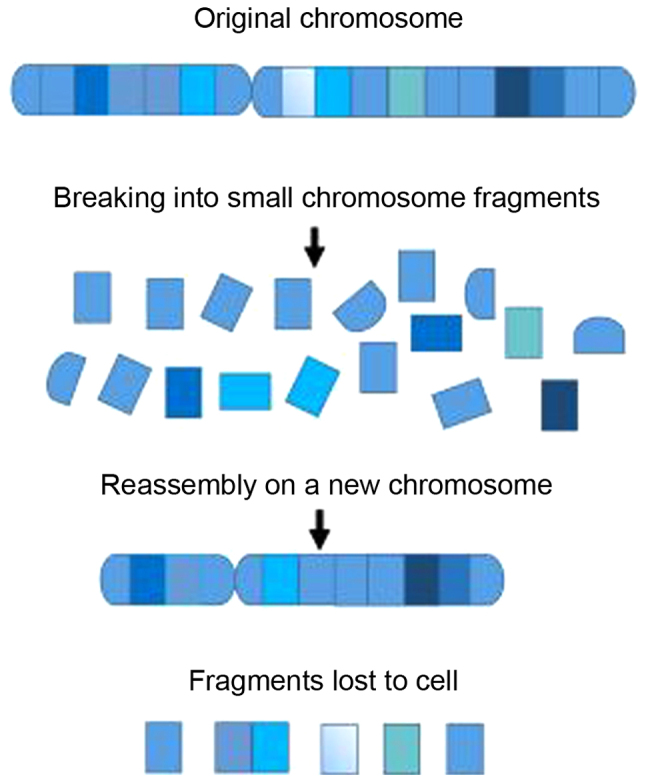
Scheme showing the process of chromothripsis in a single chromosome. Adapted with permission from ChromothripsisDB (23). Chromothripsis leads to copy number variations, namely deletions and additional structural rearrangements, such as insertions and inversions, as a result of double-stranded DNA breaks followed by nonhomologous repair.

Stephens *et al* (22) characterized a phenomenon during cancer development, which they termed chromothripsis. The chromosome or chromosomal region was segmented/broken into 10-100s of pieces, some of which were subsequently stitched back together by the endogenous DNA repair mechanisms, such as microhomology-mediated break repair and/or nonhomologous end-joining (NHEJ). The result was a mosaic patchwork of genomic fragments (22). The database ChromothripsisDB (23) is a resource for mining the existing knowledge of chromothripsis (24). Fig. 1 shows a scheme of the process of chromothripsis in a single chromosome. Ionizing radiation may contribute in part to chromothripsis (25). Mladenov *et al* (26) developed a model of double stranded break (DSB) clustering which permitted direct analysis of the consequences of determined configurations of DSB clusters in cells. Their results suggested that DSB clusters constitute the first-line DSB-processing pathways of canonical-NHEJ and homologous recombination repair. Consequently, there is an increase in the contribution of alternative end-joining and the formation of chromosomal aberrations. The authors were thus able to hypothesize a mechanism for the damage caused by high linear energy transfer radiation and the genomic rearrangements associated with chromothripsis (26). One of the mechanisms that may underlie chromothripsis involves the segmentation and breakdown of chromosomes in micronuclei where isolated chromosomes or chromosome arms undergo significant local DNA breakage and rearrangement (27). The chromosomes from ruptured micronuclei are reincorporated into daughter nuclei (28). Maciejowski *et al* (29) suggested another mechanism: Telomere fusions during telomere crisis, giving rise to anaphase bridges that persist and develop into chromatin bridges. Several steps occur after and at the end of clonal descendants derived from telomere crisis in cells displaying chromothripsis and kataegis (29). Kataegis is associated with chromothripsis (9). Chromothripsis breakpoints may result in the presence of clusters of base substitutions with close proximity (kataegis), displaying the C>T and C>G signature at TpC dinucleotides, which are associated with mutagenesis mediated by apolipoprotein B mRNA editing catalytic polypeptide-like family (29). Tubio and Estivill (30) reported that chromothripsis may be caused by aborted programmed cell death (apoptosis). Several genotoxic agents such as radiation, nutrient deprivation, infection or oxygen shortage resulted in higher-order fragmentation of chromatin and apoptosis in a cell population. However, one or even a small number of cells may not complete apoptosis and survive. These surviving cells may incorrectly repair their DNA, giving rise to rearrangements characteristic of chromothripsis (30). Using an approach based on complex alterations after selection and transformation, Mardin *et al* (31) reported an association between telomere stability and hyperploidy with chromothripsis (31). Ivkov and Bunz (32) established a model for the suppression of chromothripsis by p53, where the mutational loss of p53 gives rise to chromothripsis.

Kloosterman *et al* (33) reported evidence of local shattering of chromosomes followed by NHEJ leading to the formation of complex constitutional rearrangements involved in congenital defects, and suggested the possibility of constitutional chromothripsis (33). Analysis of 10 constitutional complex chromosomal rearrangements demonstrated that chromothripsis rearrangements may result from chromosome breakage by multiple DSBs (34). The authors found that in two patients the rearrangements were confined to a single chromosome, but in one patient multiple chromosomes were involved. These rearrangements gave rise to deletions or to copy neutral rearrangements, and clusters of DSBs were observed. The authors concluded that a common mechanism involved in chromothripsis rearrangements associated with developmental malformations may be the chromosome shattering and nonhomologous or microhomology mediated repair mechanisms (34). Nazaryan-Petersen *et al* (35) demonstrated that constitutional chromothripsis may be driven by L1-Mediated Retro-transposition and Alu/Alu Homologous Recombination. Masset *et al* (36) suggested a mechanism through which shattered chromosomes are reassembled primarily by NHEJ giving rise to complex rearranged chromosomes with or without copy-number losses, as illustrated by Fukami *et al* (37). Additionally, microhomology-mediated break-induced replication (MMBIR) may be implicated in chromothripsis (37).

The number of breaks is lower for chromothripsis rearrangements observed in the germline cells compared with chromothripsis in cancer genomes (34). These lower numbers of breaks and copy number changes in congenital chromothripsis may be due to different molecular mechanisms occurring in the development of the disease, as well as to selection in a developing embryo. It is possible that congenital chromothripsis rearrangements possess a similar architecture as simple reciprocal translocation, which, involves two breaks and subsequent formation of two derivative chromosomes (38). Constitutional chromothripsis may thus be a more complex variant of a simple reciprocal translocation (38).

The mutation rate is greater in spermatogenesis than in oogenesis (39). In the course of spermatogenesis, chromothripsis can arise due to environmental stimuli such as ionizing radiation or the generation/presence of free radicals which act as initiators of DNA damage (39). Constitutional chromothripsis may be due to an imbalance between DSB formation and repair in meiosis (40). The rearrangements observed by Kloosterman *et al* (34) were present on paternal chromosomes. This finding highlights the vulnerability of spermatogenesis to DNA damage and show that spermatogenesis is a critical stage in the genesis of congenital chromothripsis. Failure in DNA repair pathways in oocytes with the potential occurrence of abortive apoptosis or replicative stress may also trigger chromothripsis (39).

In addition to the cases of congenital disease referred to above, multiple other cases with abnormal phenotypes and constitutional chromothripsis have been described (41-43).

Chromothripsis in healthy females was described by de Pagter *et al* (44) who demonstrated that the human genome can tolerate chromothripsis rearrangements, disrupting multiple protein-coding genes with a normal phenotype. Additionally, Bertelsen *et al* (45) showed that constitutional chromothripsis may occur over several generations and was not always associated with an abnormal phenotype. Chiang *et al* (46) showed that chromothripsis occurred in the germline where it resulted in a karyotypically balanced state with chromosomal balanced abnormalities such as inversions (46).

An increasing number of reports of chromothriptic events in patients with congenital diseases (33,34,41-43), in embryos (39), but also in healthy individuals (44,45) suggest that chromothripsis is considerably more frequent than expected (7). These studies are important for the evaluation of patients with congenital diseases as well as in human health.

## 4. CNVs and constitutional chromothripsis

Clustered CNVs detected by chromosomal microarray analysis (CMA) are frequently reported as constitutional chromothripsis (47). Pettersson *et al* (48) presented two different rearrangements on chromosome 5p in a mother and her daughter, initially classified as simple CNVs. Using a combination of microarray analysis and massive parallel whole-genome sequencing, it was shown that, due to unequal crossing-over during meiosis, there was an evolution from a chromothriptic rearrangement in the mother to another complex rearrangement involving both deletions and duplications in her daughter (48). Slamova *et al* (49) studied the case of a boy with developmental and growth delay in whom karyotyping showed a seemingly balanced *de novo* complex rearrangement of 4 chromosomes. Microarray analysis detected two paternal *de novo* deletions and subsequent whole-genome mate-pair sequencing confirmed the chromothriptic nature of the rearrangement (49).

Chromothripsis results in CNVs and other structural changes (50). As referred to above, rearrangements in chromothripsis were associated with Alu/Alu NAHR. Due to the high copy number of the Alu elements, these elements are prone to NAHR events which have resulted in benign and pathogenic genomic deletions, duplications and inversions (35). Similarly, the presence of repeated sequences, such as segmental duplications or Alu sequences, were frequently observed at the break points of chromothripsis, similar to CNVs (50).

Nazaryan-Petersen *et al* (47) studied 21 clustered CNV carriers with congenital developmental disorders, intellectual disability or autism. Using whole genome sequencing to study the structures of the rearrangement first investigated by CMA, they identified a total of 83 breakpoint junctions (BPJs). Their results indicated 8 cases with deletions that frequently had additional structural rearrangements, such as insertions and inversions typical to chromothripsis, 7 cases with duplications, and 6 cases with combinations of duplications and deletions showing interspersed duplications and BPJs enriched with microhomology. Some rearrangements also indicated both a breakage-fusion-bridge cycle process and haltered formation of a ring chromosome, and 2 cases showed rearrangements mediated by Alu and long interspersed nuclear elements (LINE). The authors concluded that various mechanisms may be involved in the formation of clustered CNVs: Replication independent canonical NHEJ and alt-NHEJ, microhomology-mediated break-induced replication (MMBIR)/fork stalling and template switching, and breakage-fusion-bridge cycle and Alu- and LINE-mediated pathways. They suggested that 7 cases were chromothripsis and 10 cases were chromoanasynthesis events (47). The primary difference between chromoanasynthesis and chromothripsis is the presence of copy gains such as duplication, triplication, in addition to deletions and copy-neutral chromosomal regions (7).

Recently, Hattori *et al* (18) reported a case of a patient with transient neonatal diabetes mellitus and multiple congenital malformations who possessed a simple tandem duplication on chromosome 6q, a simple balanced inversion on chromosome 14q, two tandem inversions with a deletion on chromosome 2q, an inverted duplication with a deletion on chromosome 13q, and catastrophic rearrangements on chromosome 21q. The substantial genomic changes on chromosomes 2q and 21q were indicative of chromothripsis, and the eventual rearrangement of 13q may have also resulted from chromothripsis. The rearrangements on chromosomes 6q and 13q were are likely created initially during premeiotic mitosis in a testicular germ cell, and thereafter modified by physiological homologous recombination during meiosis I, whereas simple rearrangements on 6q and 14q are likely the result of NHEJ or replication-based errors. It is expected that these five chromosomal aberrations are not independent, but reflect a specific mutagenic event. The case in (18) had multiple *de novo* CNVs. Breakpoints of the rearrangements were indicative of replication-based errors, NHEJ and chromothripsis (18).

It thus seems possible that multiple *de novo* CNVs and constitutional chromothripsis may occur in the human genome probably as a result of the same mutagenic event. Table I summarizes the published studies on CNVs and constitutional chromothripsis and outlines the primary features and disease cases already described in the literature. These studies are important for understanding the development of the human embryo and thus in health and human disease.

## 5. Conclusion

Several studies have highlighted the importance of CNVs in human health and pathology. Likewise constitutional chromothripsis described shortly after cancer chromothripsis has become increasingly important in the study of birth defects and has also been observed in healthy subjects. Constitutional chromothripsis may coexist with multiple *de novo* CNVs. Future studies are required to further clarify the relationship between CNVs and constitutional chromothripsis. This work is of great interest not only to researchers but also to clinicians who should consider how these phenomena are involved from a clinical perspective.

For a more complete overview of chromothripsis, the book Chromothripsis. Methods and Protocols is recommended (51).

## Figures and Tables

**Table I:** Studies on copy number variations and constitutional chromothripsis.

Author, year	Phenotype	Copy number variations^a^	Chromothripsis	(Refs.)
Bertelsen *et al*, 2016	Normal	hg19xg.[chr3:[pter_135827611::137890282_138510036 inv::138510037_142218722::135827614_137735948]::chr5: 118834146_qter]; g.[chr5:pter_118834138::chr3: [137845987_137890201inv::142218723_qter]] An ~109-kb deletion on 3q22.3 was detected.	Chromothripsis transmitted through three generations in 11 healthy carriers	(45)
Pettersson *et al*, 2018	Normal	5p13.2(31820212-32131586)x1 5p13.2(36418846-36521666)x1 5p13.2(37072236-37092106)x1 5p13.2(37577701-37742275)x1 5q13.2(70150001-70220000)x1	Chromothriptic rearrangement	(48)
Pettersson *et al*, 2018	Developmental delay	5p13.2(31820212-32131586)x1 5p13.2(36521666-37072247)x3 5p13.2(37092106-37577669)x3	Complex rearrangement that evolved from a chromothriptic rearrangement in the mother	(48)
Slamova *et al*, 2018	Developmental and growth delay	Two de novo deletions of 0.7 and 2.5 Mb at two of the breakpoints in 1q24.3 and 6q24.1-q24.2, respectively	Chromothriptic rearrangement	(49)
Nazaryan-Petersen *et al*, 2018	Liver malformation	arr[GRCh37] 5p15.1(16715952_16736553x1, 16758650_16771432x1) NC_000005.9:g.[16715952_16736553del;16736554_16758649inv;16758650_16771432del]	Chromothripsis	(47)
Nazaryan-Petersen *et al*, 2018	Speech delay, autism	arr[GRCh37] 7q11.22q11.23(70610154_72399292x 1,74050199_74834365x1) dn NC_000007.14:g.[70609300_72422999del;72423000_74047984inv;74047986_74049000del]	Chromothripsis	(47)
Nazaryan-Petersen *et al*, 2018	Developmental delay, speech delay, visual abnormality, craniosynostosis	arr[GRCh37] 11q14.3 (89843044_91294308)x1 mat NC_000011.9:g.[89543002_89640782del;89640783_89766001inv;89766002_91339106del]	Chromothripsis	(47)
Nazaryan-Petersen *et al*, 2018	Speech delay, ADHD, autism	arr[GRCh37] 17p13.3(2173896_2414920)x1 pat NC_000017.10:g.[2220422_2484969del;2484970_2617882inv;2617882_2649613del]	Chromothripsis	(47)
Nazaryan-Petersen *et al*, 2018	Developmental delay, speech delay	arr[GRCh37] 21q22.3(43427355_44858483x1,45803409_48095807x1) dn NC_000021.8:g.[43414907_44797114del; 44797115_44797221inv; 44797222_45781000del; 45781001_45781001inv;45781002_ 48101999del]	Chromothripsis	(47)
Nazaryan-Petersen *et al*, 2018	Developmental delay, speech delay, growth retardation	arr[GRCh37] 4q31.3q34.1(155165258_158705411x1, 161300937_166372343x1,171349346_174403566x1) dn NC_000004.11:g.[154997276_155050346del;155164913_158707725del;158707726_171342995 inv;161297891_166374443del;171342996_174401004del]	Chromothripsis	(47)
Nazaryan-Petersen *et al*, 2018	Infantile spasms, hypotonia	arr[GRCh37] 7q11.23q21.11(75063222_77310662x1, 77629679_77770664x1,78236090_79911425x1, 82687283_82746799x1)dn NC_000007.14:g.[74942506_77216338delins[77754229_77756619inv;77770732_78236952inv;78265840_82690202inv];77226982_77226980del;77226981_77626463 inv;77626464_77626462del;77626463_78265840inv; 78265841_82754313del]	Chromothripsis	(47)
Hattori *et al*, 2019	Transient neonatal diabetes mellitus and multiple congenital malformations	46,XY,der (6) add (6)(q23.3),der (13) add (13)(q12.1),der (14) add (14)(q31),der (21) del(q11.2) add(q11.2) Two tandem inversions with a deletion on 2q Catastrophic rearrangements on 21q	Chromothripsis or chromoanasynthesis. Chromothripsis was particularly likely.	(18)

^a^Copy number variations. ADHD, attention deficit hyperactivity disorder.

## Data Availability

Not applicable.
